# Minimally invasive versus conventional sternotomy for Mitral valve repair: protocol for a multicentre randomised controlled trial (UK Mini Mitral)

**DOI:** 10.1136/bmjopen-2020-047676

**Published:** 2021-04-14

**Authors:** Rebecca H Maier, Adetayo S Kasim, Joseph Zacharias, Luke Vale, Richard Graham, Antony Walker, Grzegorz Laskawski, Ranjit Deshpande, Andrew Goodwin, Simon Kendall, Gavin J Murphy, Vipin Zamvar, Renzo Pessotto, Clinton Lloyd, Malcolm Dalrymple-Hay, Roberto Casula, Hunaid A Vohra, Franco Ciulli, Massimo Caputo, Serban Stoica, Max Baghai, Gunaratnam Niranjan, Prakash P Punjabi, Olaf Wendler, Leanne Marsay, Cristina Fernandez-Garcia, Paul Modi, Bilal H Kirmani, Mark D Pullan, Andrew D Muir, Dimitrios Pousios, Helen C Hancock, Enoch Akowuah

**Affiliations:** 1 Newcastle Clinical Trials Unit, Newcastle University, Newcastle upon Tyne, UK; 2 Department of Anthropology, Durham University, Durham, UK; 3 The Lancashire Cardiac Centre, Blackpool Teaching Hospitals NHS Foundation Trust, Blackpool, UK; 4 Health Economics Group, Population Health Sciences Institute, Newcastle University, Newcastle, UK; 5 Cardiothoracic Surgery, South Tees Hospitals NHS Foundation Trust, Middlesbrough, UK; 6 Cardiothoracic Surgery, King's College Hospital NHS Foundation Trust, London, UK; 7 Department of Cardiovascular Sciences and NIHR Leicester Biomedical Research Unit in Cardiovascular Medicine, University of Leicester, Leicester, UK; 8 Cardiothoracic Surgery, Royal Infirmary of Edinburgh, Edinburgh, UK; 9 Cardiothoracic Surgery, University Hospitals Plymouth NHS Trust, Plymouth, UK; 10 Cardiothoracic Surgery, Imperial College Healthcare NHS Trust, London, UK; 11 Cardiothoracic Surgery, University Hospitals Bristol NHS Foundation Trust, Bristol, UK; 12 Bristol Heart Institute, University of Bristol, Bristol, UK; 13 Cardiac Surgery, Liverpool Heart and Chest Hospital NHS Foundation Trust, Liverpool, UK; 14 Cardiothoracic Surgery, Liverpool Heart and Chest Hospital NHS Foundation Trust, Liverpool, UK

**Keywords:** cardiac surgery, cardiothoracic surgery, health economics

## Abstract

**Introduction:**

Numbers of patients undergoing mitral valve repair (MVr) surgery for severe mitral regurgitation have grown and will continue to rise. MVr is routinely performed via median sternotomy; however, there is a move towards less invasive surgical approaches.

There is debate within the clinical and National Health Service (NHS) commissioning community about widespread adoption of minimally invasive MVr surgery in the absence of robust research evidence; implementation requires investment in staff and infrastructure.

The UK Mini Mitral trial will provide definitive evidence comparing patient, NHS and clinical outcomes in adult patients undergoing MVr surgery. It will establish the best surgical approach for MVr, setting a standard against which emerging percutaneous techniques can be measured. Findings will inform optimisation of cost-effective practice.

**Methods and analysis:**

UK Mini Mitral is a multicentre, expertise based randomised controlled trial of minimally invasive thoracoscopically guided right minithoracotomy versus conventional sternotomy for MVr. The trial is taking place in NHS cardiothoracic centres in the UK with established minimally invasive mitral valve surgery programmes. In each centre, consenting and eligible patients are randomised to receive surgery performed by consultant surgeons who meet protocol-defined surgical expertise criteria. Patients are followed for 1 year, and consent to longer term follow-up.

Primary outcome is physical functioning 12 weeks following surgery, measured by change in Short Form Health Survey (SF-36v2) physical functioning scale. Early and 1 year echo data will be reported by a core laboratory. Estimates of key clinical and health economic outcomes will be reported up to 5 years.

The primary economic outcome is cost effectiveness, measured as incremental cost per quality-adjusted life year gained over 52 weeks following index surgery.

**Ethics and dissemination:**

A favourable opinion was given by Wales REC 6 (16/WA/0156). Trial findings will be disseminated to patients, clinicians, commissioning groups and through peer reviewed publication.

**Trial registration number:**

ISRCTN13930454.

**Strengths and limitations of this study**

- Largest randomised controlled trial of minimally invasive cardiac surgery globally.
- Strong and ongoing patient and public involvement and international clinical consensus informed trial design.
- Robust design, with 90% power, includes randomisation to surgery performed by consultant surgeons with defined expertise beyond the learning curve. Design requires some patients to move between surgeons, which may impact recruitment but assures quality of outcomes and reduces bias.
- Multicentre UK trial with blinding of central primary outcome assessor and echocardiogram core laboratory.
- Up to 5 year estimates of key clinical and survival outcomes.

## Introduction

Mitral regurgitation is most commonly caused by degenerative mitral valve disease, which leads to dilatation of the mitral valve annulus, leaflet prolapse or leaflet restriction. It can also be caused by rheumatic valve disease, or infective endocarditis.

Mitral valve repair (MVr) surgery is the optimal treatment for patients with severe mitral regurgitation caused by degenerative disease; when compared with mitral valve replacement, it carries a lower perioperative mortality, improved survival, better preservation of left ventricular function and lower long-term morbidity and mortality.^1–6^

MVr surgery for mitral valve regurgitation is frequently performed^7^ and patient numbers in the UK increased by a third between 2003 and 2012 (from 1549 to 2118^8 9^; the number of patients undergoing isolated MVr surgery more than doubled over the same time (681 in 2003 to 1456 in 2012)).^8 9^ The rise is expected to continue. Furthermore, international guidance recommends that asymptomatic patients may benefit from early surgery.^10^

### Choice of comparators

Mitral valve surgery is routinely performed via sternotomy which involves dividing the sternum completely, enabling easy access to the heart and cannulation of the great vessels centrally to establish cardiopulmonary bypass. It allows flexibility in myocardial protection strategies and potentially simplifies deairing and haemostasis at the end of the procedure.

Disadvantages of a sternotomy incision include an increase in bleeding because of the size of the incision.^11^ The mitral valve is posterior to the incision and valve access can be difficult. Wound infections occur in 2%–3% of patients, and significant morbidity, and mortality, can result from this complication.^12 13^ The sternotomy incision is usually closed using multiple stainless steel wires facilitating immobilisation of the sternum while sternal union occurs. It can take up to 3 months for the sternum to heal completely.^12^ During this period, the activity of patients is significantly limited to reduce the risk of sternal dehiscence. These limitations can reduce the speed of recovery and limit patients’ ability to return to usual activities. Moreover, limited activity during this period can increase the risk of complications in the recovery period.

Minimally invasive approaches are increasingly used in all surgical specialties. In cardiac surgery, they are routinely used for coronary revascularisation^14^ aortic valve surgery, aortic root surgery^15^ and surgery for assist devices.^16^

Several publications have established the safety of minimally invasive mitral valve surgery.^17–22^ Outcomes in a cohort of 1000 patients undergoing minimally invasive mitral valve surgery between 2003 and 2011 demonstrated a mortality of 0.7% at 1 year, and 8 year survival of 90%.^18^ Of those surviving, freedom from reoperation at 8 years was 93%.^18^ The approach may also significantly reduce morbidity and mortality in high-risk patients including in the elderly.^19–21^ A propensity matched analysis of 2400 cases from 3 UK centres suggested a reduction of blood transfusion (20.5% vs 14.4%, p=0.005) and median length of hospital stay (7 vs 6 days, p<0.001) when minimally invasive surgery was compared with conventional surgery despite longer procedural times.^22^

There is emerging evidence from non-NHS healthcare settings that the minimally invasive approach is less costly than conventional sternotomy; with cost savings driven by reduced intensive care and hospital stay as well as reduced need for blood transfusion.^23^

### Current evidence supporting the rationale for the trial

Five published meta-analyses compare minimally invasive with conventional MVr.^24–28^ These identify only two small randomised controlled trials (RCTs) reporting short-term^29^ and long-term^30^ outcomes and base their main conclusions on evidence from observational studies;^18 19 23 31–38^ these provide no evidence of differences in clinical outcomes. The literature is severely limited by the absence of robust data on comparative outcomes between the two techniques.^24–28^

The International Society of Minimally Invasive Cardiac surgery published a consensus document on the role of minimally invasive mitral valve surgery in contemporary cardiac surgery practice in 2010.^39^ This highlights the limitations of available evidence in reaching a consensus, particularly the lack of adequately powered prospective RCTs to establish the comparative effectiveness of the two approaches. Its recommendation was for further prospective RCTs, adequately powered to assess quality of life, complications, efficacy (repair rates) and clinically relevant outcomes, particularly long term survival, patient functionality and freedom from re-operation.^39^

Less than 5% of patients having mitral valve surgery in the UK currently have a minimally invasive MVr, largely as a result of the lack of clear and definitive evidence. There is consensus in the cardiac surgery community that the optimum surgical approach to treat these patients urgently needs defining. This is particularly pressing as there is emerging evidence relating to the effectiveness of percutaneous MVr with the mitraclip and other devices in degenerative^40^ and functional mitral regurgitation.^41^ It is now clear that trials of percutaneous approaches and surgery are needed, but cannot be undertaken until the optimal surgical approach is clearly defined.

## Methods and analysis

### Study design

UK Mini Mitral is a multicentre, RCT of minimally invasive thoracoscopically-guided right minithoracotomy (intervention) versus conventional sternotomy (control) in patients undergoing MVr. The trial includes an internal pilot to assess the likelihood of meeting recruitment targets.

The trial will answer the questions ‘Are improvements in physical functioning and associated return to usual activities seen in patients who undergo minimally invasive mitral valve surgery compared with conventional surgery?’ and ‘Is minimally invasive mitral valve surgery compared with conventional surgery cost-effective?’

### Setting

Three hundred and sixteen patients due to receive MVr surgery will be recruited from UK NHS cardiothoracic surgery units with established minimally invasive and conventional mitral valve services. All centres will be able to accommodate the needs of this trial including established minimally invasive MVr services, research nurse and echocardiographer support, and facilities to carry out trial assessments. Only units in equipoise in the way they manage their patients requiring MVr will participate.

At each centre, individual surgeons perform one type of operation; MVr via thoracoscopically guided right minithoracotomy or MVr via median sternotomy. Before performing surgery within the trial, each surgeon has completed 50 of these; the Trial Steering Committee (TSC) reviews a record of the number of operations for each surgeon, and agrees to their participation, in advance of them doing so. Depending on their allocation, patients can be required to move to another surgeon following randomisation.

### Eligibility criteria

Patients are eligible to take part if they are scheduled to have surgery for MVr and fulfil all eligibility criteria:

#### Inclusion criteria

- Adult (≥18 years old at consent) with degenerative mitral valve disease, requiring MVr*.
- Written informed consent.
- Fit for cardiac surgery and cardiopulmonary bypass.

*Patients requiring concomitant surgery for atrial fibrillation (AF), tricuspid valve repair, and/or patent foramen ovale (PFO) closure are included

#### Exclusion criteria

- Concomitant cardiac surgery other than for AF, tricuspid valve repair and PFO closure.
- Requiring mitral valve replacement.
- Acute (current) infective endocarditis.
- Emergency or salvage surgery.
- Only one surgical technique indicated.
- Pregnant.**
- Currently participating in another interventional clinical trial.
- ≥ 4 weeks as an inpatient prior to randomisation.
- Previous cardiac surgery.
- Mobility impairments that would preclude Short Form Health Survey (SF36-v2) completion.

**Female patients between the ages of 18 and 50 will receive a pregnancy test at baseline.

### Randomisation

Eligible patients will be randomised in a 1:1 ratio to undergo MVr using minimally invasive thoracoscopically guided right minithoracotomy (intervention under study) or conventional median sternotomy (control arm/usual care) by members of the research team at each centre using a 24-hour, central, secure, web-based randomisation system with concealed allocation (procured from Sealed Envelope).

Randomisation is performed using a minimisation scheme, which adjusts for baseline SF-36v2 physical functioning score, presence or absence of AF and/or PFO, and presence and severity of tricuspid regurgitation.

### Trial surgical interventions

The intervention arm will receive MVr via minimally invasive thoracoscopically guided right minithoracotomy. The control group receive MVr via conventional sternotomy.

### Intervention arm

The patient is intubated with a single or double lumen endotracheal tube. Cardiopulmonary bypass is established by femoral artery cannulation and venous return achieved from the venae cavae using a single bicaval cannula placed from the femoral vein, or with an additional cannula in the superior venae cava. Transoesophageal echocardiography confirms the optimum location of the venous and arterial cannulas.

A 4–7 cm right lateral minithoracotomy is then used to enter the thorax through the third or fourth intercostal space. A soft-tissue retractor, with or without a small thoracic retractor, is used to spread the ribs. The pericardium is opened 3–4 cm anterior and parallel to the phrenic nerve from the distal ascending aorta to the diaphragm. A video camera is inserted through a 5–10 mm port.

Endoballoon occlusion, or a transthoracic clamp, achieves aortic occlusion. Cardiac arrest is achieved with repeated doses of cardioplegia. The mitral valve is approached through a paraseptal incision and a left atrial retractor is used to expose the mitral valve.

Following the mitral valve procedure, the left atrium is closed, the heart deaired and aortic occlusion removed. Cardiopulmonary bypass is then discontinued and the thoracotomy incision closed once haemostasis has been achieved.

### Control arm

The sternum is divided completely (from the collarbone to the bottom of the breastbone) and cardiopulmonary bypass established by siting cannulas in the right atrium and inferior venae cava and ascending aorta.

Cardiac arrest is achieved with cardioplegia and the mitral valve approached via the left atrium. The valve is repaired and assessed intraoperatively by water testing. Once the repair is deemed satisfactory, the atrium is closed, deairing manoeuvres performed, and the aortic cross clamp removed to allow reperfusion of the heart. Cardiopulmonary bypass is discontinued once haemostasis is achieved and the sternum is closed.

### Outcomes

The primary outcome is physical functioning and associated return to usual activities measured by change in SF-36v2 physical functioning scale^42^ 12 weeks following index surgery using a 4-week recall period.

The primary economic outcome is cost effectiveness measured in terms of incremental cost per quality-adjusted life year (QALY) gained over 52 weeks following index surgery.

Secondary outcomes post index MVr:

- Feasibility and study recruitment, explored by means of a 6-month internal pilot.
- Cardiac function; assessed using transthoracic echocardiography (TTE), early (1 day to 12 weeks) and late (52 weeks) by an independent core laboratory.
- Mitral valve and mitral valve surgery related events and survival (morbidity and mortality); compared at 52 weeks and up to 5 years using adverse event data and Hospital Episode Statistics and National Institute for Cardiac Outcomes Research data.
- Physical functioning and quality of life over 52 weeks; compared using SF-36v2 and EuroQol 5 Dimension 5 Level (EQ-5D-5L).^42–45^
- Levels of physical activity and quality of sleep; quantified using accelerometer measures (GENEActiv^46–50^) over 52 weeks.
- Surgical outcomes quantified over 52 weeks.
- Costs, including intervention-specific estimates, of the two operations and their sequalae over 52 weeks.
- QALYs, estimated from EQ-5D-5L^43–45^ and SF-6D (derived from the SF-36v2) over 52 weeks.
- Modelled costs and QALYs over the patient lifetime.
- Modelled incremental cost per QALY gained over the patient lifetime
- Hospital Episode Statistics (HES) data will be explored to understand if it adequately captures mitral valve events.

### Sample size

The primary outcome measure is change in physical functioning scale within SF-36v2^42^ from baseline to 12 weeks following index surgery. On the basis of the literature and consulting with cardiac and patient communities, a minimally clinically important difference is 10 points on the scale. UK Mini Mitral is powered to investigate the superiority of thoracoscopically guided right minithoracotomy over conventional sternotomy.

One study^31^ reports a small variation (SD of 8) in SF-36 physical function scores; this is not seen in other studies^32 33^ which report a SD of 30. We used the conservative estimate to inform the original sample size calculation:

Assuming alpha of 5% and 90% power, 382 patients (191 in each arm) would be required to detect a minimally clinically important difference of 10 points in the SF-36v2^42^ physical functioning scale at 12 weeks (assuming SD of 30); allowing for attrition, we planned to randomise 400 patients. To assess our assumptions, we performed a blinded sample size re-estimation using baseline SF-36v2 physical functioning scale data from 177 trial patients. Using the re-estimated SD of 26.3 with 90% power, 288 patients are required to detect a 10 point difference in SF-36v2 physical functioning at 12 weeks. Accounting for 10% attrition, 316 patients will be recruited.

### Trial procedures

**Figure 1:**
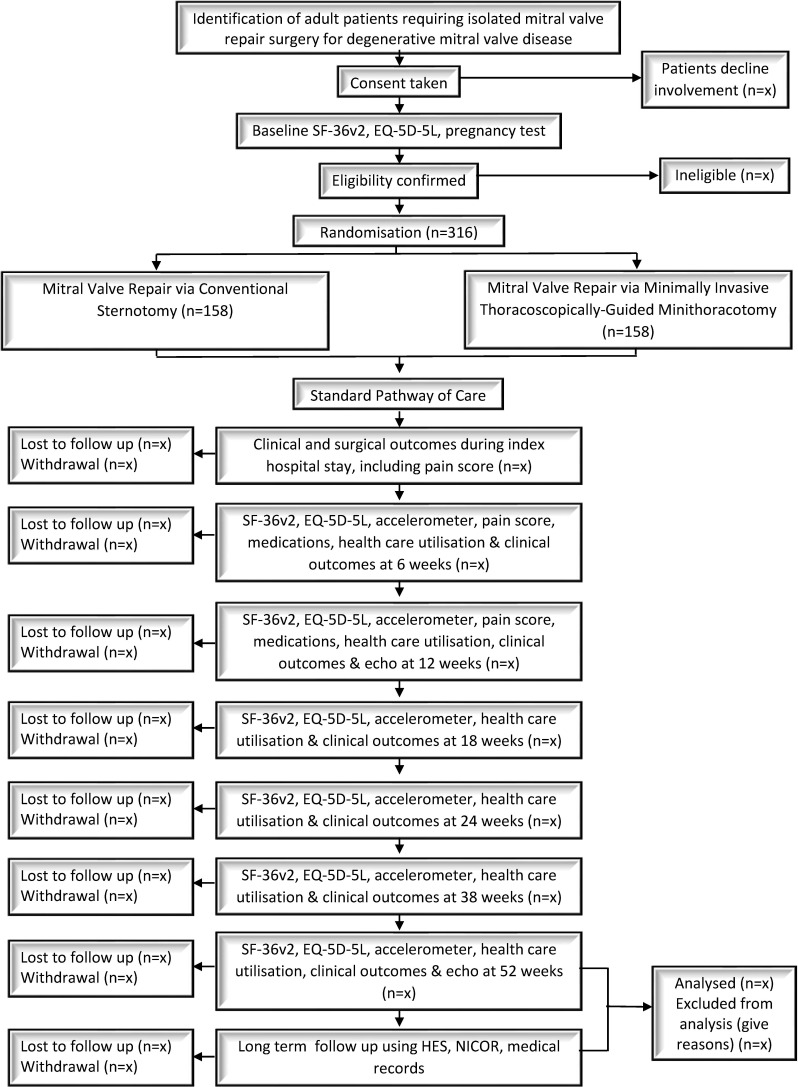
UK Mini Mitral Trial flow diagram. EQ-5D-5L, EuroQol 5 Dimension 5 Level; SF-36v2, Short Form Health Survey.

Patients due to undergo isolated MVr surgery at participating centres are identified at the point of referral (elective patients) and from the inpatient waiting list (urgent but non-emergency patients) by the clinical research team and approached about the trial, including given an information sheet and consent form. After discussion, consent is sought, baseline assessments performed and eligibility checked and confirmed by one of the consultant surgeons participating in the trial. Eligible patients are randomised, and their general practitioners informed. A full schedule of events is detailed in table 1, and a participant flow chart provided in figure 1. Patients have assessments at 6, 12, 18, 24, 38 and 52 weeks following index surgery. Longer term follow-up will involve routinely collected data.

**Table 1:** UK Mini Mitral Schedule of Events

Study procedure	Baseline	Day of surgery	Index hospital stay	Follow-up: time is calculated from the day of index surgery					
	−26 weeks to day of surgery	Day 0	Day 0 until discharge following index surgery	6 weeks	12 weeks	18 weeks	24 weeks	38 weeks	52 weks
Consent	X								
Medical history	X								
Physical examination	X								
Demographics	X								
Concomitant medications	X			X	X				X
Pregnancy test	X								
NYHA class	X			X	X				
ECHO (TTE)	X		X	X	X				X
TOE	X	X							
SF-36v2	X			X	X	X	X	X	X
EQ-5D-5L	X			X	X	X	X	X	X
euroSCORE		X							
Accelerometer	X			X	X	X	X	X	X
Eligibility check	X								
Randomisation	X								
MVr surgery		X							
Postoperative details			X						
Wound Pain score			X^i^	X	X				
Ward usage and date of discharge			X						
Discharge destination			X						
RBC and other blood product transfusions		X	X						
AEs and SAEs		X	X	X	X	X	X	X	X
Reoperation/further surgery		X	X	X	X	X	X	X	X
Health Care Utilisation Questionnaire				X	X	X	X	X	X
HES data				X	X	X	X	X	X
NICOR data				X	X	X	X	X	X
Medical record review				X	X	X	X	X	X

AE, adverse event; ECHO, Echocardiogram; EQ-5D-5L, EuroQol 5 Dimension 5 Level; HES, Hospital Episode Statistics; MVr, mitral valve repair; NICOR, National Institute for Cardiac Outcomes Research; NYHA, New York Heart Association; RBC, Red Blood Celleuor; SAE, serious adverse event; SCORE, European System for Cardiac Operative Risk Evaluation; SF36-v2, Short Form Health Survey; TOE, transoesophageal echocardiography; TTE, transthoracic echocardiography.

### SF-36v2 and EQ-5D-5L

Physical functioning and overall quality of life are assessed using SF-36v2 and EQ-5D-5L^42–45 51^ questionnaires administered prior to randomisation. Questionnaire completion is repeated over the telephone at 6, 12, 18, 24, 38 and 52 weeks following index surgery.

### Echocardiogram

Cardiac function is assessed via TTE at baseline, post operatively (1 day to 12 weeks) and at 52 weeks. Measurements of left ventricular volumes, dimensions and function, mitral regurgitation severity and estimates of right heart function and pulmonary artery pressure are reported.

### Accelerometer

Levels of physical activity and quality of sleep are quantified using accelerometer measures. Accelerometers are worn on patients’ non-dominant wrist nonstop for 7 consecutive days at seven timepoints (baseline, 6, 12, 18, 24, 38 and 52 weeks following index surgery). The restrictions of movement by being an inpatient means accelerometer measures are not possible for inhouse urgent patients and are not to be undertaken.

### Statistical analysis

Primary analysis will follow intention to treat principles, with patients analysed according to randomisation, irrespective of surgical intervention received. A full statistical analysis plan will be developed and agreed with the Independent Data Monitoring and Ethics Committee (IDMEC) and TSC prior to the end of data collection.

Outcome data will be analysed at the end of the study; no interim outcome analyses are planned.

Primary analysis of change in physical functioning, measured using SF-36v2 at 12 weeks, will use a general linear model accounting for surgical intervention, baseline scores, valve pathology and concomitant surgery. Robust SE will account for intrapatient correlation due to repeated measures. A similar modelling approach will be used for the final analysis of change in physical functioning at 52 weeks.

Secondary outcomes: continuous outcomes will be analysed using a general linear model, binary outcomes will be analysed using a generalised estimating equation to account for repeated binary data over time, categorical outcomes (with more than two categories) will be analysed using baseline-category logit model, while log-rank test and frailty modelling will be used to analyse time-to-event outcomes. Analysis of all outcomes with repeated measures on the same patient will account for intrapatient correlation. Multiple imputation for secondary outcomes will be used if necessary to sensitise incomplete data. All outcomes will also be described using simple statistics to facilitate interpretation and communication of findings. Secondary analysis will also include hierarchical modelling of patients, surgeons and centres, to provide an estimate of treatment effect at each of these levels.

Each question within the SF-36v2 physical functioning scale will be scored based on the RAND scoring system.^42^ The average of the per-patient scale scores (assuming each question carries equal weight) will provide the primary outcome for physical functioning at 12 weeks.

### Economic analysis

The economic evaluation will include both a within trial economic evaluation to estimate the incremental cost per QALY gained at 52 weeks and a model based economic evaluation to estimate cumulative costs and QALYs and incremental cost per QALY gained over a patient lifetime. The analysis will take the perspective of the UK NHS.

The within trial analysis will consist of a microcosting of the intervention (minimally invasive minithoracotomy) and usual care (conventional sternotomy). The cost of each surgical procedure will be derived from information captured in the case report form and obtained from the team at clinical centres. The subsequent use of NHS services will be captured through patient questionnaires to record use of subsequent primary and secondary care services.

A state transition model will enable extrapolation of costs and outcomes beyond 52 weeks for the predicted lifetime of participants. Data used to populate the model will come from the within trial analysis and external data sources systematically derived from the literature as per best practice guidance.^52^ External data will help identify health state utilities for events occurring long term and not captured within the trial. This will include HES data, validated using medical records and the National Adult Cardiac Surgical Database, adjudicated by an independent expert panel. The independent expert panel will assess which use of health services is attributable to sequelae from index surgery, or underlying disease condition (eg, surgical revision or complication, worsening mitral disease or infection) and/or which is unrelated.

### Trial conduct and governance

The Trial Management Group oversees all day-to-day aspects to ensure that the protocol is adhered to, ensuring patient safety and data integrity; they meet monthly. The IDMEC report to the TSC and provide advice on the ongoing conduct and safety; they meet 6 monthly. The TSC, where independent members are in the majority, provides overall supervision; they meet 6 monthly.

### Patient and public involvement

Meetings with cardiac surgeons and cardiologists in the UK and the USA established the need for, and feasibility of, a definitive trial. Ideas were discussed with patient members of the South Cleveland Heart Fund in 2014 and again in 2015. All patients fully supported the trial and informed the primary outcome; their view that returning to usual activities was the best way to compare the two techniques matched that of surgeons and cardiologists. A patient is a member of the TSC and the team have committed to sharing the results of this trial with patients.

### Blinding

SF-36v2 and EQ-5D-5L assessments are completed over the telephone by a central blinded assessor.

All echocardiograms are sent to an independent Core Laboratory for assessment by a senior echocardiographer, who reports these without knowledge of the intervention.

## Ethics and dissemination

The trial team carefully thought through potential issues and aimed to address these in the trial design. This study does not include patients who will lack capacity, nor minors. This trial is not based in an emergency setting. The trial is conducted in accordance with the protocol, the principles of Good Clinical Practice, and the favourable ethical opinion. All patients provide written informed consent prior to participation, and for their data to be used in future research.

The main issue is a relatively new cardiac procedure, though all cardiac surgery has inherent risks. To mitigate this, surgery will only be performed by highly experienced consultant cardiac surgeons who will have performed at least 50 operations of the same type prior to the start of their involvement. The trial will require the collection and storage of sensitive personal data. All data are handled in accordance with the appropriate legislation. We will only publish deidentified aggregated data.

The results of the trial are expected to fill an important and large gap in the international literature. We expect that the trial results will inform an important revision of national and international. We will publish the findings in peer-reviewed journals, and disseminate results to patients, and the international clinical community.

## Supplementary Material

Reviewer comments
