# Supplementary material for: Minimally invasive versus conventional sternotomy for Mitral valve repair: protocol for a multicentre randomised controlled trial (UK Mini Mitral)
Source: BMJ Open. 2021 Apr 14;11(4):e047676. doi: 10.1136/bmjopen-2020-047676 (PMC8054102; doi:10.1136/bmjopen-2020-047676)
Supplement: Reviewer comments [file bmjopen-2020-047676.reviewer_comments.pdf]

PEER REVIEW HISTORY

BMJ Open publishes all reviews undertaken for accepted manuscripts. Reviewers are asked to complete a checklist review form (**http://bmjopen.bmj.com/site/about/resources/checklist.pdf**) and are provided with free text boxes to elaborate on their assessment. These free text comments are reproduced below.

ARTICLE DETAILS

**TITLE (PROVISIONAL)**	Minimally invasive versus conventional sternotomy for mitral valve repair: protocol for a multi-centre randomised controlled trial (UK Mini Mitral)
**AUTHORS**	Maier, Rebecca; Kasim, Adetayo; Zacharias, Joseph; Vale, Luke; Graham, Richard; Walker, Antony; Laskawski, Grzegorz; Deshpande, Ranjit; Goodwin, Andrew; Kendall, Simon; Murphy, Gavin; Zamvar, Vipin; Pessotto, Renzo; Lloyd, Clinton; Dalrymple-Hay, Malcolm; Casula, Roberto; Vohra, Hunaid; Ciulli, Franco; Caputo, Massimo; Stoica, Serban; Baghai, Max; Niranjan, Gunaratnam; Punjabi, Prakash; Wendler, Olaf; Marsay, Leanne; Fernandez-Garcia, Cristina; Modi, Paul; Kirmani, Bilal; Pullan, Mark; Muir, Andrew; Pousios, Dimitrios; Hancock, Helen; Akowuah, Enoch

This article was not externally reviewed at BMJ Open. Protocols that have been independently assessed prior to submission to BMJ Open are usually fast-tracked to publication on the grounds that further substantial changes will not be possible. This independent assessment will usually be external, independent review for both a substantial grant award from a non-commercial or government funder and ethics approval.
